# Evaluation of macrocyclic hydroxyisophthalamide ligands as chelators for zirconium-89

**DOI:** 10.1371/journal.pone.0178767

**Published:** 2017-06-02

**Authors:** Nikunj B. Bhatt, Darpan N. Pandya, Jide Xu, David Tatum, Darren Magda, Thaddeus J. Wadas

**Affiliations:** 1 Department of Cancer Biology, Wake Forest School of Medicine, Winston-Salem, North Carolina, United States of America; 2 Lumiphore, Inc., Berkeley, California, United States of America; Genentech Inc, UNITED STATES

## Abstract

The development of bifunctional chelators (BFCs) for zirconium-89 immuno-PET applications is an area of active research. Herein we report the synthesis and evaluation of octadentate hydroxyisophthalamide ligands (**1** and **2**) as zirconium-89 chelators. While both radiometal complexes could be prepared quantitatively and with excellent specific activity, preparation of ^89^Zr-**1** required elevated temperature and an increased reaction time. ^89^Zr-**1** was more stable than ^89^Zr-**2** when challenged *in vitro* by excess DTPA or serum proteins and *in vivo* during acute biodistribution studies. Differences in radiometal complex stability arise from structural changes between the two ligand systems, and suggest further ligand optimization is necessary to enhance ^89^Zr chelation.

## Introduction

The development of zirconium-89 (^89^Zr: t_1/2_ = 78.4 h; β^+^; 23%, 909 keV)-based PET radiopharmaceuticals has intensified since comprehensive procedures for producing ^89^Zr in high specific activity and attaching it to monoclonal antibodies (mAbs) were first described [1–3]. The half-life and positron emission properties of ^89^Zr make it ideal for the labeling of mAbs, which require extended circulation time for effective *in vivo* antigen targeting. Several ^89^Zr-labeled mAbs are currently in clinical trials [4–6].

The most widely used bifunctional chelators (BFCs) for ^89^Zr chelation are desferrioxamine (DFO) and its analogues, which are derivatives of the iron chelator desferral, a siderophore secreted by *Streptomyces pilosus* [7,8]. Although DFO contains three hydroxamate groups, which seem to provide effective chelation for ^89^Zr *in vitro*, this leaves 2 empty coordination sites for the 8-coordinate ^89^Zr and published reports described elevated bone retention in murine models receiving injections of ^89^Zr-DFO-labeled agents [9,10]. Several papers have attempted to explain this phenomenon through molecular modeling and experimental studies [11,12]. Further investigations have attempted to enhance the stability of the ^89^Zr-DFO complex or design new ligands as BFCs for ^89^Zr [13–24].

**Fig 1:**
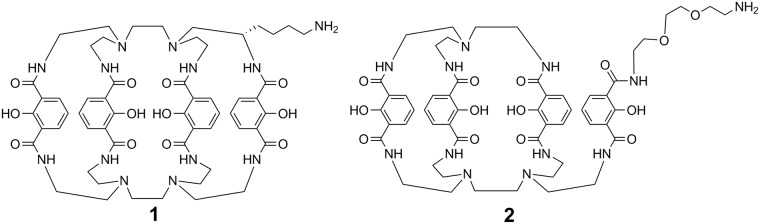
BFCs 1 and 2. These ligands contain 2-hydroxyisophthalamide units for octadentate coordination of Zr^4+^ and a pendant arm containing a primary amine, which can be easily functionalized for conjugation to a variety of targeting ligands.

In this report, we describe the development of BFCs containing four 2-hydroxyisophthalamide coordinating units for ^89^Zr chelation (Fig 1). We became interested in hydroxyisophthalamide (IAM) based-ligands, since these coordination motifs are similar to those used by bacteria for metal ion sequestration and have been used successfully as chelators for a variety of lanthanide metal cations [25–32]. Additionally, they offer potential advantages for ^89^Zr BFC design [33]. These aromatic systems bind through a combination of phenolic and carbonyl oxygen atoms, which should aid coordination to hard radiometals such as zirconium. Furthermore, the IAM unit can be easily derivatized to quickly expand the diversity of ligands that can be synthesized and evaluated.

## Materials and methods

Zirconium-89 (^89^Zr: t_½_ = 78.4 h, β^+^: 22.8%, E_β+max_ = 901 keV; EC: 77%, E_γ_ = 909 keV) was purchased from Washington University School of Medicine (St. Louis, MO) or Zevacor, Inc. (Dulles, VA). Unless otherwise noted, all other chemicals were purchased from Sigma-Aldrich Chemical Co. (St. Louis, MO), and solutions were prepared using ultrapure water (18 MΩ-cm resistivity). Radiochemistry reaction progress and purity were analyzed using a Waters analytical HPLC (Milford, MA), which runs Empower software and is configured with a 1525 binary pump, 2707 autosampler, 2998 photodiode array detector, 2475 multichannel fluorescence detector, 1500 column heater, fraction collector, Grace Vydac 218MS C18 column (5 μm, 4.6 × 250 mm, Grace Davidson, DeerField, IL) and a Carrol Ramsey 105-s radioactivity detector (Berkeley, CA). All ligands (**1** and **2**) and associated ^Nat^Zr-complexes were monitored at 220 nm using a mobile phase consisting of 0.01% TFA/H_2_O (solvent A) and 0.01% TFA/acetonitrile (solvent B), and a gradient consisting of 0% B to 70% B in 20 min at a flow rate of 1.2 mL/min. In addition, radio-TLC was conducted on a Bioscan AR 2000 radio-TLC scanner equipped with a 10% methane:argon gas supply and a PC interface running Winscan v.3 analysis software (Eckert & Ziegler, Berlin, DE). Varian ITLC-SG strips were employed using a 50 mM DTPA (pH 7) solution as eluent, and the complex ^89^Zr(ox)_2_ as a standard control. Radioactive samples were counted using a Perkin Elmer 2480 Wizard^®^ gamma counter (Waltham, MA). Preparation of ligand **1** has been reported [27]. Starting compound **3**, which was used for the synthesis of **2**, was synthesized as described previously [32].

### Synthesis

#### N,N',N",N‴-[1,2-ethanediylbis(nitrilodi-2,1-ethanediyl)]tetrakis{2-benzyloxy-3-[(2-thioxo-3-thiazolidinyl)carbonyl]benzamide} (5)

Tetrakis-(2-aminoethyl) ethylene diamine **4** (581 mg, 2.50 mmol) was dissolved in ca. 10% isopropyl alcohol in dichloromethane (48 mL) and triethylamine (1.74 mL, 12.5 mmol) and added using a syringe pump (NE1000) to a solution of 2-benzyloxy-1,3-phenylenebis((2-thioxothiazolidin-3-yl)methanone) **3** (23.7 g, 50 mmol) in dichloromethane (anhydrous, 100 mL) and triethylamine (1.74 mL, 12.5 mmol) over 50 h at a rate of 1.00 mL/hr. After a further 24 h, solvent was removed under reduced pressure, and the crude product was purified by silica gel chromatography using 0.1% triethylamine, 2–5% isopropyl alcohol in dichloromethane as eluents. Fractions containing product were combined, solvent was removed under reduced pressure, and the residue dried *in vacuo* to provide compound **5** (1.611 g, 39.0%). ^1^H NMR (300 MHz, CDCl_3_): δ = 8.02–7.99 (d of d, 4H, ArH), 7.46–7.44 (m, 8H, ArH), 7.30–7.20 (m, 20H, PhH), 4.96 (s, 8H, PhCH_2_O), 4.39 (t, 8H, NCH_2_CH_2_S), 3.26 (m, 8H, CH_2_NC = O), 3.02 (t, 8H, NCH_2_CH_2_S), 2.38 (m, 12H, CH_2_N). ^13^C NMR (300 MHz, CDCl_3_): δ = 201, 167, 165, 154, 136, 134, 132, 130, 129, 128, 125, 59, 55, 53, 52, 38, 29. FTMS pESI: calculated for C_82_H_81_N_10_O_12_S_8_ [MH]^+^, 1653.3796, found, 1653.3855.

#### Benzyl-protected bi-macrocycle (7)

A solution of tris-(2-aminoethyl)amine **6** (54 mg, 365 μmol) in isopropyl alcohol (50 mL) and triethylamine (255 μL) and a solution of N,N',N",N‴-[1,2-ethanediylbis(nitrilodi-2,1-ethanediyl)]tetrakis[2-benzyloxy-3-[(2-thioxo-3-thiazolidinyl) carbonyl]benzamide **5** (213 mg, 528 μmol) in dichloromethane (50 mL) were added dropwise to a solution of dichloromethane (1.50 L) and triethylamine (255 μL), degassed three times with N_2_, over 4 days using two syringe pumps at 0.5 mL/hr. After an additional day of reaction, solvent was removed under reduced pressure, and the crude product was purified by silica gel chromatography using 0.1% triethylamine, 2–7.5% isopropyl alcohol in dichloromethane as eluents. Fractions containing product were combined, solvent was removed under reduced pressure, and the residue dried *in vacuo* to provide the protected bi-macrocycle **7** (167 mg, 31.7%). ^1^H NMR (300 MHz, CDCl_3_): δ = 7.94–6.74 (broad m, 32H, PhH, ArH), 5.00 (s, 2H, PhCH_2_O), 4.80–4.75 (br s, 6H, PhCH_2_O), 4.48 (t, 2H, NCH_2_CH_2_S), 3.50–3.32 (m, 14H, CH_2_NC = O), 3.17 (t, 2H, NCH_2_CH_2_S), 2.64–2.26 (m, 18H, CH_2_N). ^13^C NMR (600 MHz, CDCl_3_): δ = 201.6, 167.3, 167.0, 166.6, 166.3, 165.5, 164.7, 154.5,153.3, 153.2, 136.0, 135.1, 134.1, 133.2, 132.7, 131.7, 130.3, 129.4, 129.2, 129.1, 128.8, 128.6, 128.4, 127.7, 127.5, 124.6, 124.4, 79.0, 78.4, 77.7, 72.5, 71.6, 71.2, 70.4, 70.1, 61.8, 55.7, 55.3, 55.0, 54.2, 52.9, 52.4, 51.9, 51.2, 50.2, 48.8, 39.1, 38.3, 37.8, 37.5, 31.6, 29.6, 28.7, 26.6, 19.2, 13.9. FTMS pESI: calculated for C_79_H_84_N_11_O_12_S_2_ [M+H]^+^, 1442.5737, found, 1442.5753.

#### Benzyl and *tert*-butyloxycarbonyl-protected bi-macrocycle (9)

A solution of benzyl-protected bi-macrocycle **7** (154 mg, 108 μmol) in dry dichloromethane (5 mL) and triethylamine (74 μL) was treated with N-Boc-2,2’-(ethylenedioxy)diethylamine **8** (40 mg, 161 μmol) under N_2_, and stirred for 28 h. Solvent was removed under reduced pressure, and the crude product was purified by silica gel chromatography using 0.1% triethylamine, 3.5–5% methanol in dichloromethane as eluents. Fractions containing product were combined, solvent was removed under reduced pressure, and the residue dried *in vacuo* to provide the protected bi-macrocycle **9** (148 mg, 87%). ^1^H NMR (300 MHz, MeOD): δ = 7.8–6.9 (broad m, 32H, PhH, ArH), 5.5 (s, 2H, PhCH_2_O), 3.6–3.1 (m, 26H, CH_2_CH_2_O, CH_2_NC = O), 2.7–2.4 (m, 18H, CH_2_N), 1.4 (s, 9H, CH_3_). ^13^C NMR (300 MHz, MeOD): δ = 154.4, 153.7, 136.5, 132.5, 132.0, 129.4, 129.0, 128.6, 124.7, 124.5, 79.2, 78.5, 78.1, 70.2, 69.2, 53.9, 50.0, 40.2, 39.8, 39.1, 38.3, 38.0, 27.8. FTMS pESI: calculated for C_87_H_103_N_12_O_16_ [M+H]^+^, 1571.7610, found, 1571.7667.

#### Bi-macrocyclic bifunctional chelator (2)

Benzyl and *tert*-butyloxycarbonyl-protected bi-macrocycle **9** (89 mg, 57 μmol) was dissolved in 12N hydrochloric acid (1.5 mL) and glacial acetic acid (1.5 mL). The solution was stirred under an inert atmosphere for 26 h, whereupon HCl was removed with a stream of inert gas. Solvents were removed under reduced pressure and the residue was dried *in vacuo*. The residue was dissolved in methanol (2000 μL) and transferred to four O-ring microcentrifuge tubes. Ether (ca. 1.5 mL) was added, and the tubes were placed at 4°C for 1 h. The tubes were centrifuged at 12,000 rpm for 3 min and decanted. Pellets were washed with ether (ca. 1.5 mL), allowed to air dry, and then dried *in vacuo* to provide bi-macrocycle **2**, tetrahydrochloride salt (58.6 mg, 82%). ^1^H NMR (500 MHz, CD_3_OD, 25°C): *δ* 8.18–7.43 (m, 8H), 7.05–6.48 (m, 4H), 4.36–3.23 (m, 42H), 2.97 (s, 2H). ^13^C NMR (100 MHz, CDCl3, 25°C): *δ* 120.11, 71.52, 71.49, 70.50, 40.80, 40.56 (note: broad signals due to macrocyclic structure impeded full characterization of the ^13^C resonances). FTMS pESI: calculated for C_54_H_71_N_12_O_14_ [M+H]^+^, 1111.5207, found, 1111.5234. Anal: Calculated (found) C_54_H_84_N_12_O_19_Cl_4_, 48.13 (48.15), 6.29 (6.22), 12.48 (12.11).

#### Zr-1

Compound **1** (6.9 mg, 5.1 mmol) was dissolved into methanol (200 μL), and Zr(acac)_4_ (1.94 mg, 3.98 mmol) was separately dissolved into methanol (200 μL). The solutions were combined and allowed to sit at room temperature for 1 hour. Triethylamine (1.5 mL) was added, causing a white precipitate to form. The precipitate was isolated by centrifugation, and the supernatant was removed. The solid residue was then dissolved into methanol (400 μL) and again precipitated by adding triethylamine (1.5 mL). The product was dissolved and precipitated in this way a total of 3 times, and then the residual solvent was removed under vacuum, yielding a white solid of Zr-**1**. Yield: 3.78 mg, 66%. ^1^H NMR (500 MHz, CD_3_OD, 25°C): *δ* 7.81–7.23 (m, 8H), 6.54–6.08 (m, 4H), 3.42–3.12 (m, 42H), 2.77 (s, 2H). HRMS-ESI (*m/z*, [M+H]^+^) Calcd for C_56_H_70_N_13_O_12_^90^Zr: 1206.4308, Found: 1206.4348. Anal. Calcd (Found) for ZrC_56_H_69_N_13_O_12_·1.5C_6_H_16_NCl·1.5H_2_O: C 54.18 (54.22), H 6.72 (6.69), N 14.09 (14.06) %.

#### Zr-2

Compound **2** (5.5 mg, 4.1 mmol) was dissolved into methanol (200 μL), and Zr(acac)_4_ (1.44 mg, 2.95 mmol) was separately dissolved into methanol (200 μL). The solutions were combined and allowed to sit at room temperature for 1 hour. Triethylamine (1.5 mL) was added, causing a white precipitate to form. The precipitate was isolated by centrifugation, and the supernatant was removed. The solid residue was then dissolved into methanol (400 μL) and again precipitated by adding triethylamine (1.5 mL). The product was dissolved and precipitated in this way a total of 3 times, and then the residual solvent was removed under vacuum, yielding a white solid of Zr-**2**. Yield: 2.83 mg, 71%. ^1^H NMR (500 MHz, CD_3_OD, 25°C): *δ* 7.91–7.43 (m, 8H), 6.48–6.02 (m, 4H), 3.62–3.12 (m, 42H), 2.85 (s, 2H). HRMS-ESI (*m/z*, [M+H]^+^) Calcd for C_54_H_67_N_12_O_14_^90^Zr: 1197.3947, Found: 1197.4004. Anal. Calcd (Found) for ZrC_54_H_66_N_12_O_14_·2C_6_H_16_NCl·H_2_O: C 53.14 (53.24), H 6.76 (6.67), N 13.15 (13.03) %.

### Density functional theory calculations

Ground state density functional theory calculations were performed at the Molecular Graphics and Computational Facility, College of Chemistry, University of California, Berkeley using Gaussian 09 [34]. The ground state geometries of Zr**-1** and Zr**-2** (S3 Fig and S1 Table) were optimized using the B3LYP functional, treating the light atoms (H through O) with the 6-31G(d,p) basis set [35–38]. The Zr atom was treated with the effective core potential MWB28 [39,40]. No solvent, symmetry constraints, or counterions were included in the calculations. Crystal structures of the related H22 linked 2-hydroxyisophthalamide (IAM) ligands bound to Tb^III^ were used as starting points for these Zr^IV^ complexes [27]. In addition to geometry optimizations, frequency calculations were performed, and all structures are presented in energy minimized conformations.

### Radiolabeling of macrocyclic 2-hydroxyisophthalamide ligands (1, 2) with ^89^Zr

The complexation of ^89^Zr with macrocyclic 2-hydroxyisophthalamide ligands (**1**, **2**) was achieved by reacting 5–10 μg (5–10 μL,1.0 mg/mL in water) of each ligand with an aliquot of ^89^Zr(ox)_2_ (0.6 mCi, 22.2 MBq) diluted in 100 μL of water and pH adjusted to 7–7.5 using 1 M Na_2_CO_3_. The ^89^Zr-**1** reaction was incubated at 95°C for 2 h; the ^89^Zr-**2** reaction was incubated at 50°C for 1 h. Formation of ^89^Zr-**1** and ^89^Zr-**2** was monitored by radio-TLC using Varian ITLC-SG strips and 50 mM DTPA (pH 7) as the mobile phase. In this system, unchelated ^89^Zr forms a complex with DTPA and elutes near the solvent front (denoted as the red line at 130 mm in radio-TLC chromatograms); the ^89^Zr-ligand complex remains at the origin (denoted as the red line at 30 mm in radio-TLC chromatograms) (S4 Fig). The identity of each radioactive complex was further confirmed by comparing its radio-HPLC elution profile to the UV-HPLC spectrum of its nonradioactive Zr-complex (S5 and S6 Figs).

### Determination of partition coefficients (log P)

The partition coefficient (log P) for each complex was determined by adding 5 μL of each ^89^Zr-labeled complex (5 μCi; 0.19 MBq) to a mixture of 500 μL of octanol and 500 μL of water [41]. The resulting solutions (n = 8) were vigorously vortexed for 5 min at room temperature and then centrifuged for 5 min to ensure complete layer separation. From each sample, a 50 μL aliquot was removed from each phase. Radioactivity in each phase was counted separately in a gamma counter. Each organic phase was washed with water before gamma counting. The partition coefficient was calculated as a ratio of counts in the octanol fraction to counts in the water fraction. The log P values were reported as an average of four measurements (S2 Table).

### *In vitro* serum stability and DTPA challenge study

*In vitro* stability was carried out by adding 10 μL of each ^89^Zr-labeled complex (50 μCi, 1.85 MBq) to 500 μL DTPA (50 mM, pH7), or human serum (S3 Table). The solutions (n = 3) were incubated at room temperature and 37°C, respectively. Samples were analyzed daily for 7 days using radio-TLC (Varian ITLC-SG strips and 50 mM DTPA (pH 7)) and gamma counting (energy window: 500–1500 keV) [9].

### Animal model

All studies were carried out in strict accordance with the recommendations in the Guide for the Care and Use of Animals of the National Institutes of Health, and approved by the Wake Forest University Health Sciences Institutional Animal Care and Use Committee (Protocol # A14-081). Normal mice used in the experiments were received from The Jackson Laboratory, USA and were accommodated in the housing room operated according to the Animal Welfare Act, the guidelines established by the NIH and all other Federal and State statutes and regulations pertaining to laboratory animal care. All animals were acclimated to a 12-hour light/dark cycle and received food and water *ad libitum*. All animals were checked by veterinary staff daily to ensure well-being, proper environment and monitor for signs of distress. Clinical signs of distress in laboratory rodents include decreased activity, pilo-erection, un-groomed appearance, excessive licking and scratching, self-mutilation, abnormal stance, hunched appearance, rapid or shallow respiration, grunting, dilated pupils, aggressiveness towards handler, high pitch vocalizations, change in feeding activity, and attempts to separate from group. In the event of clinical distress, animals were euthanized after consulting the on-call veterinarian. To minimize suffering during the tail vain injection, animals were kept under deep isoflurane anesthesia. At the end point of the study, animals were anesthetized with isoflurane and were euthanized by cervical dislocation, which is consistent with the recommendations of the American Veterinary Medical Association guidelines on Euthanasia. Animals were monitored every 30 minutes during the experiment, but during the experiment, none of the animals died or were found ill before experimental end point.

### Biodistribution studies

Biodistribution studies were based upon a previously published procedure [42]. Briefly, female NIH Swiss mice (6–8 wk old, n = 6) were injected with each ^89^Zr-labeled complex (0.55 MBq (15 μCi)/mouse) via the tail vein, and sacrificed at 2, 4, 24, 48 and 72 h post-injection. Organs and tissues of interest were excised and weighed, and radioactivity was counted on a gamma counter. The percent injected dose per gram (%ID/g) was calculated by comparison to a weighed, counted standard for each group (S5 and S6 Tables).

### Statistical methods

All of the data are presented as mean±SD or mean (95% confidence intervals). For statistical classification a Student’s t test (two-tailed, unpaired) or one-way anova (with Tukey’s multiple comparison post-test) was performed using GraphPad Prism software (San Diego, CA). p<0.05 was considered significant.

## Results and discussion

Ligand **1** was synthesized according to published procedures [27]. Preparation of an IAM bi-macrocyclic ligand **2** (Scheme 1) began with 2-benzyloxy-1,3-phenylenebis((2-thioxothiazolidin-3-yl)methanone) **3**, which was condensed with tetrakis-(2-aminoethyl) ethylene diamine **4** under pseudo-first order conditions to provide the activated tetra-amide **5**. This was reacted with tris-(2-aminoethyl)amine **6** under high dilution conditions to form the bi-macrocycle **7**. The remaining activated amide in **7** was reacted with amine **8** to provide bi-macrocycle **9**. Protective groups were removed using a solution of concentrated hydrochloric acid in acetic acid to provide bi-macrocycle **2**. Both ligand **1** and ligand **2** were completely characterized and observed using NMR spectroscopy, high resolution mass spectrometry and elemental analysis. The nonradioactive Zr-**1** and Zr-**2** complexes were prepared by reacting ligands (**1** and **2**, 1 equiv. each) with Zr(acac)_4_ (0.75 equiv.) in methanol. Characterization of both complexes by NMR revealed spectra containing a complex pattern of multiplets that could not be resolved further. However, high resolution mass spectrometry (S1 and S2 Figs) and elemental analysis confirm the identity of each complex. Interestingly, several peaks were observed in the UV-HPLC chromatograms (S5 and S6 Figs), but based upon mass spectrometry and elemental analysis, it is believed that these peaks are structural isomers that form upon the coordination of the Zr^4+^ ion in solution. It is not implausible to imagine the formation of several structural isomers, which are dependent upon the number of phenolic and carbonyl oxygen atoms involved in metal ion complexation. Attempts were made to isolate each isomer by HPLC for NMR analysis, but isomeric inter-conversion was observed during this process, which made our attempts at further characterization unsuccessful. While attempts to elucidate the molecular structure of Zr-IAM complexes using single crystal x-ray diffraction analysis are ongoing, we performed density functional theory calculations on both Zr-**1** and Zr-**2** to model ground state coordination geometries (S1 Table). If we neglect the alkyl amine linker arm, the structure of Zr-**1** (S3 Fig) has twofold rotational symmetry, where the symmetry axis passes through the midpoint of the central ethylene units and through the metal center. Modeling revealed the energy minimized structure of Zr**-2** could exist with two distinct binding modes (A or B; S3 Fig), although both are quite similar. In structure A, the amine sidearm is connected to the amide not involved in Zr^4+^ ion coordination. In structure B, the amine sidearm is connected to the amide directly involved in Zr^4+^ ion coordination. Despite these differences, structure A was found to be only 0.42 kcal/mol lower in energy than structure B, which is a very small difference given the size and flexibility of the ligands. Thus it is likely that both A and B are present at significant quantities in solution, which further suggests that the multiple peaks observed in the HPLC chromatograms result from coordination isomers in solution.

**Scheme 1**. **Synthesis of bi-macrocyclic bifunctional chelator 2**.

**Scheme 2**. **Radiochemical synthesis of** ^**89**^**Zr-1 and** ^**89**^**Zr-2**. Proposed structures of ^89^Zr-1 and ^89^Zr-2 are depicted, but other structural isomers may be possible.

Our initial radiochemistry studies with **1** revealed that elevated reaction time and temperature were necessary for quantitative radiolabeling (S4 Fig). Although quantitative radiolabeling was achieved, these conditions would be too harsh for radiolabeling of **1**-mAb conjugates. Accordingly, we reduced the structural rigidity of **1** by partially uncoupling one of the isophthalamide coordinating units to yield **2**. Using this strategy, the time and temperature needed to quantitatively radiolabel **2** with ^89^Zr (S4 Fig) decreased markedly. Furthermore, an A_s_ of 0.7 GBq/μmol was observed, which is comparable to the A_s_ of similar ^89^Zr-complexes reported in the literature [14,19,23].

Lipophilicity (log P) plays an essential role in estimating the biodistribution of ^89^Zr-complexes *in vivo* [41]. Using the water-octanol partition method, the log P (pH 7) of ^89^Zr-**1** and ^89^Zr-**2** was determined to be -2.97±0.02 and -1.45±0.06, respectively, compared to -2.83±0.04 for ^89^Zr-DFO (S2 Table). These negative values suggest that the complexes possess hydrophilic character, which is expected to result from the abundant hydrogen bonding motifs displayed by the radiometal complexes while in solution. These values are similar to recently published values for ^89^Zr-terepthalamide and ^89^Zr-hydroxypyridinone complexes [14,19,23]. Complexes of **1** and **2** also contain 4 and 3 tertiary amines in the scaffold, respectively, in addition to the primary amines, some of which might be protonated at neutral pH.

Initial stability studies were also conducted by incubating ^89^Zr-**1** or ^89^Zr-**2** in the presence of a 50 mM DTPA solution over 7 days (S3 Table). ^89^Zr-**1** was more resistant to DTPA challenge than ^89^Zr-**2**. Only 15% and 28% of ^89^Zr-**1** underwent transchelation after 1 and 7 days, respectively, while 37% and 74% of ^89^Zr-**2** underwent transchelation during the same study period. ^89^Zr-**DFO** displayed stability similar to ^89^Zr-**2** under these conditions, with 45% and 59% transchelation after 1 and 7 days, respectively. Likewise, incubation in human serum revealed ^89^Zr-**1** was more resistant to serum protein challenge than ^89^Zr-**2** (S4 Table). While 75% of ^89^Zr-**1** remained intact after 7 days, only 17% of ^89^Zr-**2** was observed at the same time point. ^89^Zr-**DFO** remained intact upon incubation with serum. The resistance of ^89^Zr-**1** vs. ^89^Zr-**DFO** to DTPA challenge may arise from the enhanced binding that occurs through the four IAM units, which can accommodate the ^89^Zr^4+^ ion to form an eight coordinate complex. On the other hand, reduced stability of ^89^Zr-**2**, observed as ^89^Zr^4+^ ion transchelation to DTPA or serum proteins, may be caused by the reduced structural rigidity of ligand **2**, which results in dissociation of an IAM coordinating unit and subsequent loss of the coordinated ^89^Zr^4+^ ion.

**Fig 2:**
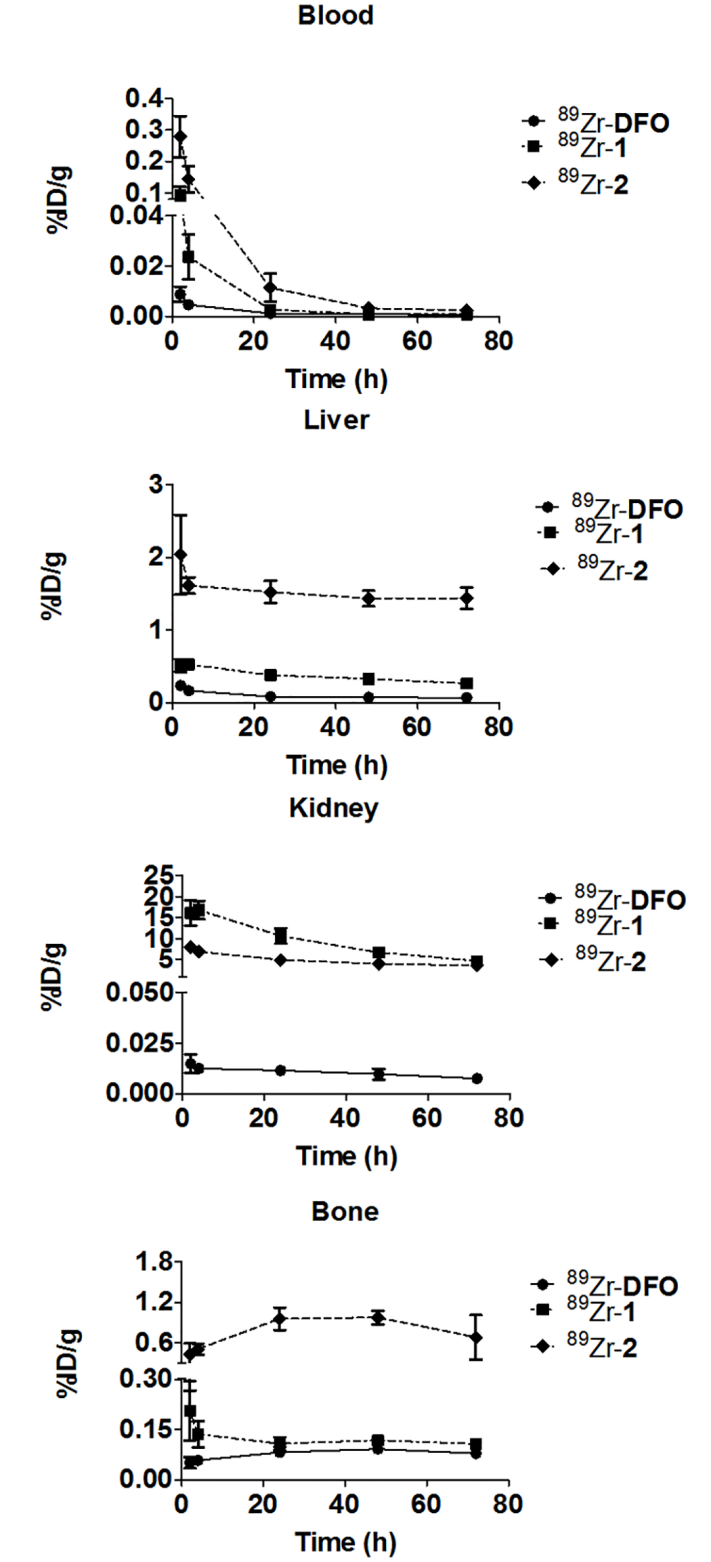
Biodistribution and clearance of ^89^Zr-DFO, ^89^Zr-1, and ^89^Zr-2 from selected tissues. ^89^Zr-**DFO** demonstrated a more favorable biodistribution than ^89^Zr-**1** and ^89^Zr-**2**. ^89^Zr-**DFO** data is taken from reference 19.

^89^Zr-**1** and ^89^Zr-**2** were evaluated *in vivo* through biodistribution studies in normal mice. Fig 2 displays the clearance properties of ^89^Zr-**1**, ^89^Zr-**2** and ^89^Zr-**DFO** from the blood, liver, kidney, and bone; and complete biodistribution details can be found in SI (S5–S7 Tables). Animals injected with ^89^Zr-**1** and ^89^Zr-**2** demonstrated elevated levels of radioactivity in the blood at early time points when compared to animals injected with ^89^Zr-**DFO** [19]. However by 72 h p.i., blood retention was comparable in animals receiving ^89^Zr-**DFO** and ^89^Zr-**1**, but not ^89^Zr-**2**. (^89^Zr-**DFO** vs. ^89^Zr-**1** vs. ^89^Zr-**2**: (mean%ID/g±SD; [one-way ANOVA]: 0.00033±0.0008 vs. 0.0008±0.0008 vs. 0.002±0.001; [F(2,15) = 40.23, p < 0.0001]). These differences, especially at earlier time points, may relate to the different ligand systems used to chelate ^89^Zr.

**DFO** is a hexadentate acyclic ligand that forms a +1 charged complex. Conversely, **1** is a trimacrocycle comprised of 24 and 30-atom rings, whereas **2** forms a bi-macrocyclic complex of 24 and 27 membered rings; both ligands are octadentate (Fig 1). The altered charge of ^89^Zr-**1** and ^89^Zr-**2** when compared to ^89^Zr-**DFO** might enhance their interactions with serum proteins, which retard clearance from the blood stream. These observations correlate well with the *in vitro* serum stability data obtained for both ^89^Zr-IAM complexes.

By 72 h p.i., the initial amount of radioactivity in liver tissue of mice receiving ^89^Zr-**DFO**, ^89^Zr-**1** or ^89^Zr-**2** decreased by 72%, 49%, and 29%, respectively. Animals injected with ^89^Zr-**DFO** had the least radioactivity in liver tissue by the end of this study, while liver tissue of animals injected with ^89^Zr-**2** had roughly 6 fold more activity than in mice receiving ^89^Zr-**1**. (^89^Zr-**DFO** vs. ^89^Zr-**1** vs. ^89^Zr-**2**: (mean%ID/g±SD; [one-way ANOVA value]: 0.066±0.009 vs. 0.26±0.019 vs. 1.43±0.15; [F(2,15) = 445.0, p < 0.0001]). Since **2** is a more flexible structure compared to ligand **1**, ^89^Zr in complex with **2** most likely undergoes facile transchelation within the liver. Considering that radioactivity was not cleared during this experiment, we surmise that the ^89^Zr formed an insoluble precipitate within the liver tissue, and is most likely ^89^Zr[Zr]-phosphate, which has been shown to be retained there without excretion [11].

Animals receiving ^89^Zr-**DFO** had significantly less radioactivity in kidney tissue when compared to the ^89^Zr-IAM complexes at every time point. By 72 h p.i., animals receiving ^89^Zr-**DFO**, ^89^Zr-**1** or ^89^Zr-**2** had excreted 49%, 71%, and 54% of the activity recorded at 2 h (^89^Zr-**DFO** vs ^89^Zr-**1** vs. ^89^Zr-**2**: (mean%ID/g±SD; [one-way ANOVA value]: 0.69±0.098 vs. 4.71±0.84 vs. 3.60±0.79; [F(2,15) = 82.11, p < 0.0001]). As the ^89^Zr-IAM complexes diffuse from the blood pool into the kidney, pH may decrease, which can cause changes in the charge or structure of these radiometal complexes. These changes may induce aggregation or transchelation, which contribute to the retention of radioactivity in kidneys. Initially, less radioactivity was observed in the kidneys of mice injected with ^89^Zr-**2**; however, the amounts for both IAM complexes become similar within 48 hours, consistent with the slower plasma clearance of ^89^Zr-**2**. Further study is needed to ascertain whether increased radioactivity in the kidney relative to **DFO** would occur using antibody-chelator conjugates.

Since bone marrow is often considered a dose-limiting organ, radioactivity retention in bone was also examined [11]. Animals injected with either ^89^Zr-IAM complex demonstrated significantly more radioactivity in their bone tissue when compared to animals injected with ^89^Zr-**DFO** at 72 h p.i. (^89^Zr-**DFO** vs ^89^Zr-**1** vs. ^89^Zr-**2**: (mean%ID/g±SD; [one-way ANOVA value]: 0.079±0.014 vs. 0.11±0.006 vs. 0.68±0.33; [F(2,15) = 17.57, p = 0.0001]). Interestingly, the biodistribution trends for ^89^Zr-**1** and ^89^Zr-**2** are very different. The amount of radioactivity in the bone of animals that received ^89^Zr-**1** diminished over time, and may reflect perfusion and clearance kinetics rather than demetallation and incorporation into the bone matrix. A similar phenomenon has been observed with a recently described hydroxypyridinone ligand [14]. By contrast, radioactivity in the bones of mice receiving ^89^Zr-**2** continually increased during this experiment, and most likely reflects radiometal chelate instability, transchelation, and metabolic sequestration of the ^89^Zr within the phosphate-rich bone matrix.

## Conclusions

Herein, we describe the synthesis and evaluation of hydroxyisophthalamide ligands as bifunctional chelators for ^89^Zr using log P, DTPA, and serum challenge studies. Additionally, the biodistribution of ^89^Zr-**1** and ^89^Zr-**2** was evaluated in normal mice. While the preparation of ^89^Zr-**1** required higher temperatures and longer reaction times compared to ^89^Zr-**2**, it was more resistant to exogenous ligand challenge. Animals injected with ^89^Zr-**1** retained less radioactivity in the blood, liver, and bone than those receiving ^89^Zr-**2**. These differences are believed to be caused by increased flexibility engineered into ligand **2**. These data suggest that IAM ligands, which were previously found useful for the sequestration of a variety of lanthanide metal cations, can also function as ^89^Zr chelators. Additional ligands that synergize the facile radiochemistry of **2** with the enhanced stability demonstrated by ^89^Zr-**1** are currently being evaluated in ^89^Zr-immuno-PET applications, and will be reported in due course.

## Supporting information

S1 Scheme Synthesis of Zr-1. (PDF)

S2 Scheme Synthesis of Zr-2. (PDF)

S1 Fig ESI-MS analysis of Zr-1. (PDF)

S2 Fig ESI-MS analysis of Zr-2. (PDF)

S3 Fig Energy minimized structures of Zr-1 and Zr-2. (PDF)

S4 Fig Radio-ITLC analysis of ^89^Zr(Ox)_2_ (A), ^89^Zr-1 (B) and ^89^Zr-2 (C) using 50mM DTPA (pH 7) as eluent. (PDF)

S5 Fig Radio-HPLC of ^89^Zr-1. (PDF)

S6 Fig Radio-HPLC of ^89^Zr-2. (PDF)

S1 Table DFT coordinates for the energy minimized structures of Zr-1 and Zr-2. (PDF)

S2 Table Log P values for ^89^Zr-complexes. (PDF)

S3 Table Stability of ^89^Zr-complexes in 50 mM DTPA (pH 7). (PDF)

S4 Table Stability (% intact) of ^89^Zr-complexes in human serum at 37°C. (PDF)

S5 Table Biodistribution data of ^89^Zr-1 in normal mice. (PDF)

S6 Table Biodistribution data of ^89^Zr-2 in normal mice. (PDF)

S7 Table Biodistribution data of ^89^Zr-DFO in normal mice. (PDF)
