# Supplementary figures and images for: Evaluation of macrocyclic hydroxyisophthalamide ligands as chelators for zirconium-89
Source: PLoS One. 2017 Jun 2;12(6):e0178767. doi: 10.1371/journal.pone.0178767 (PMC5456358; doi:10.1371/journal.pone.0178767)

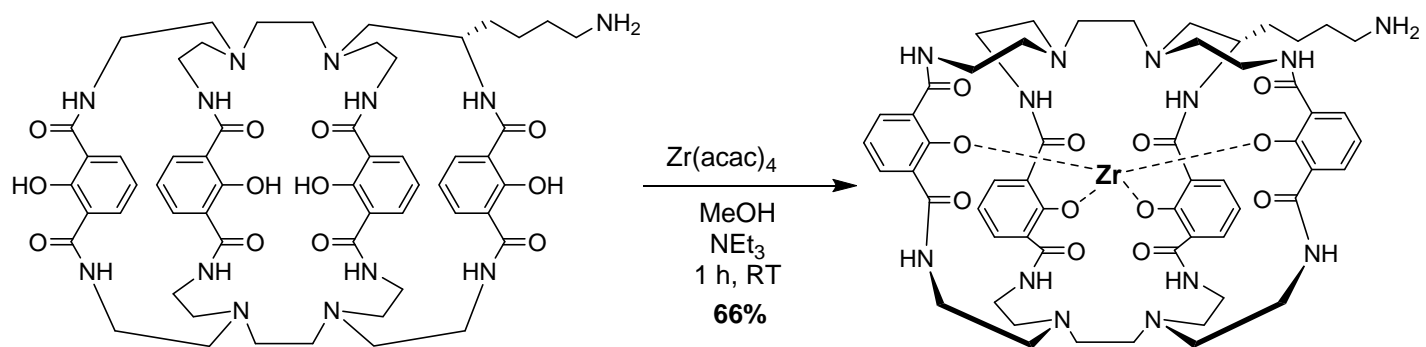

Supplement: S1 Scheme — (PDF) [file pone.0178767.s001.pdf]

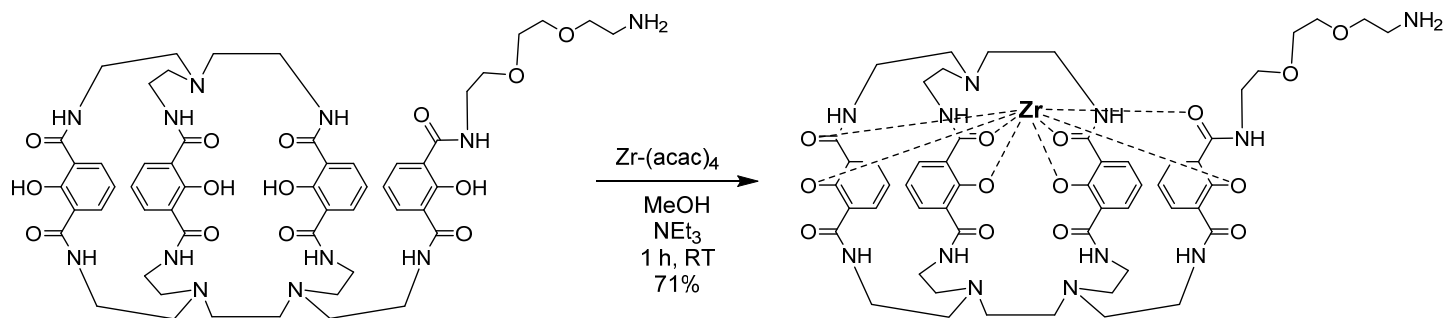

Supplement: S2 Scheme — (PDF) [file pone.0178767.s002.pdf]

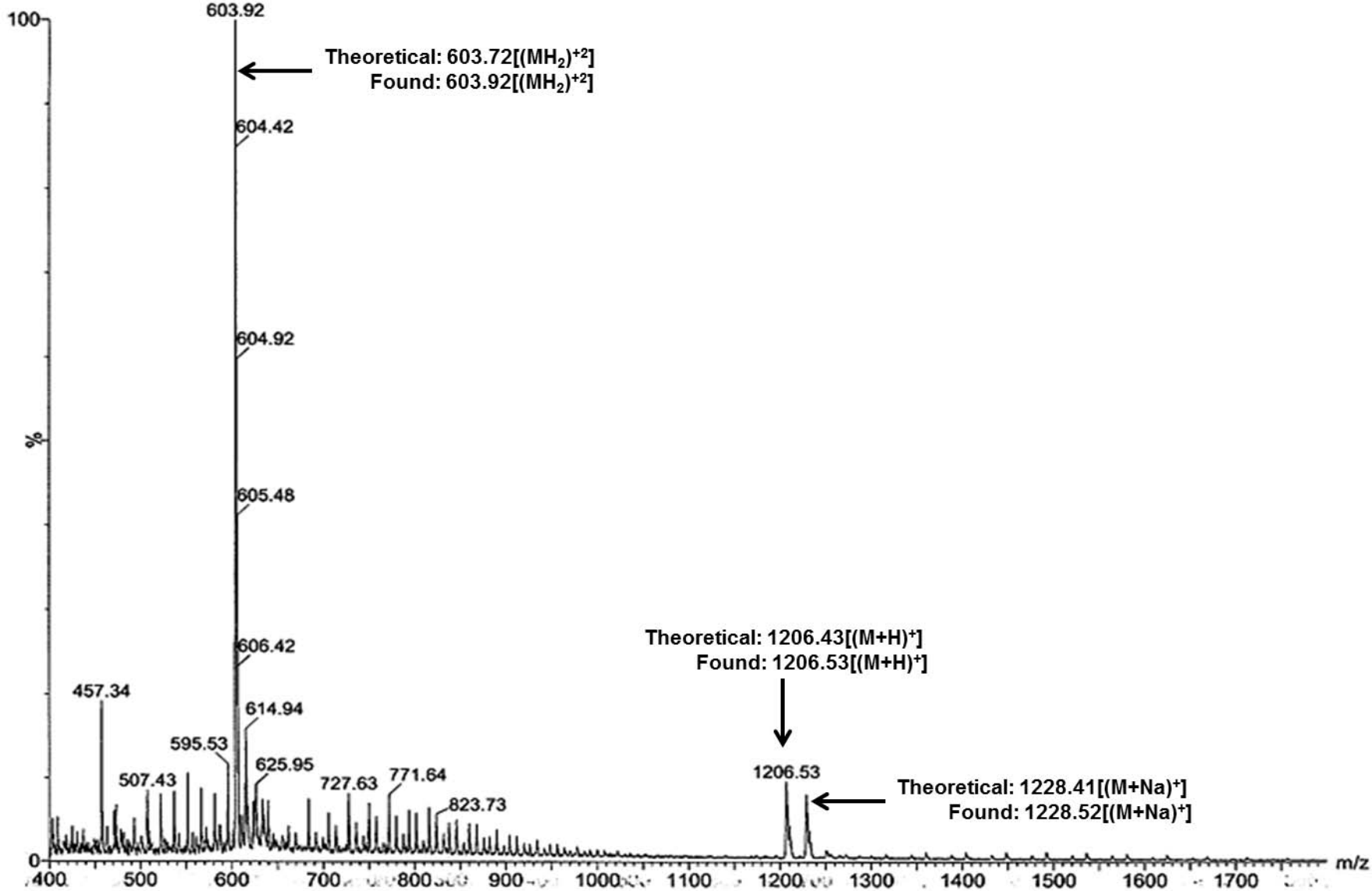

Supplement: S1 Fig — (PDF) [file pone.0178767.s003.pdf]

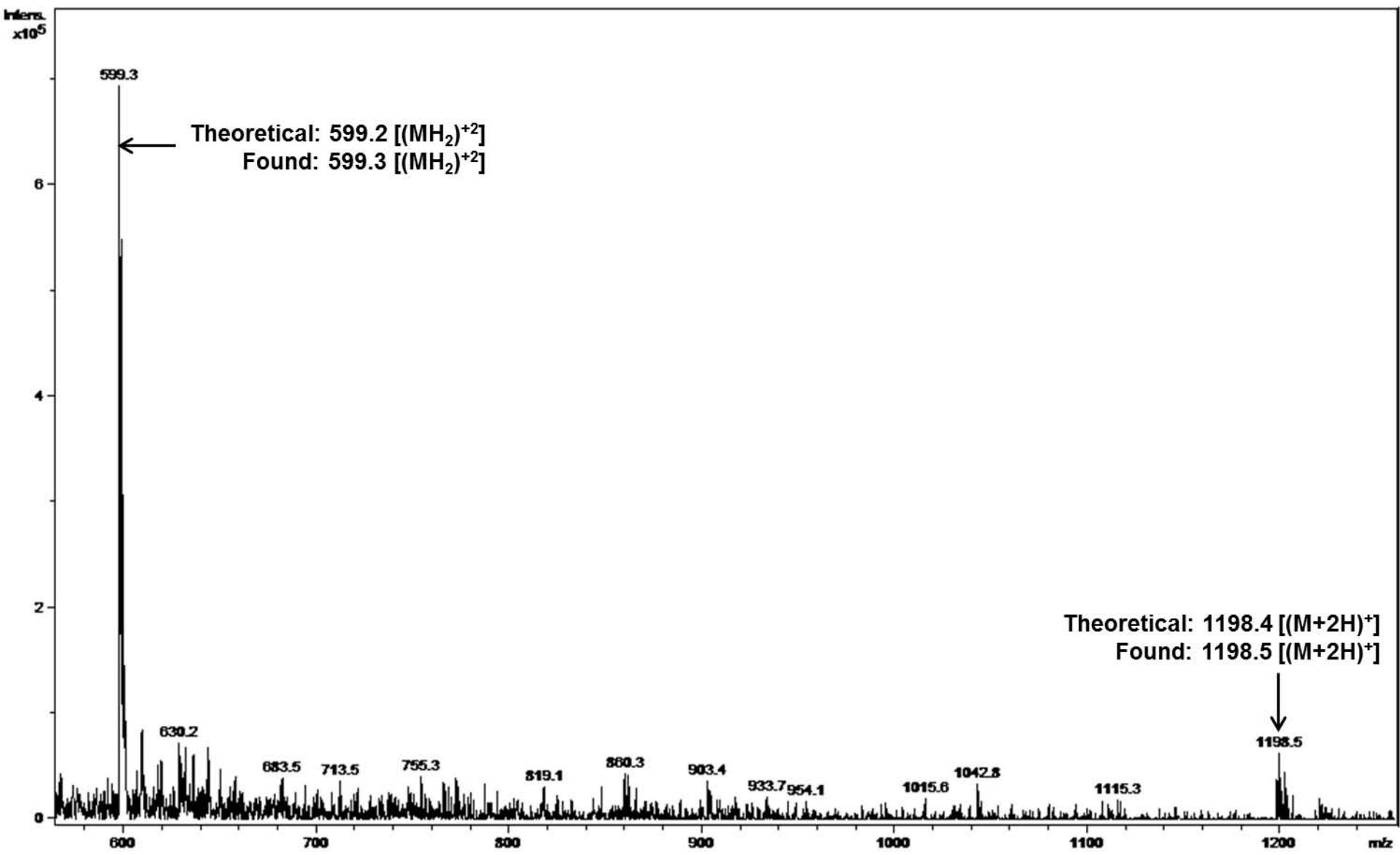

Supplement: S2 Fig — (PDF) [file pone.0178767.s004.pdf]

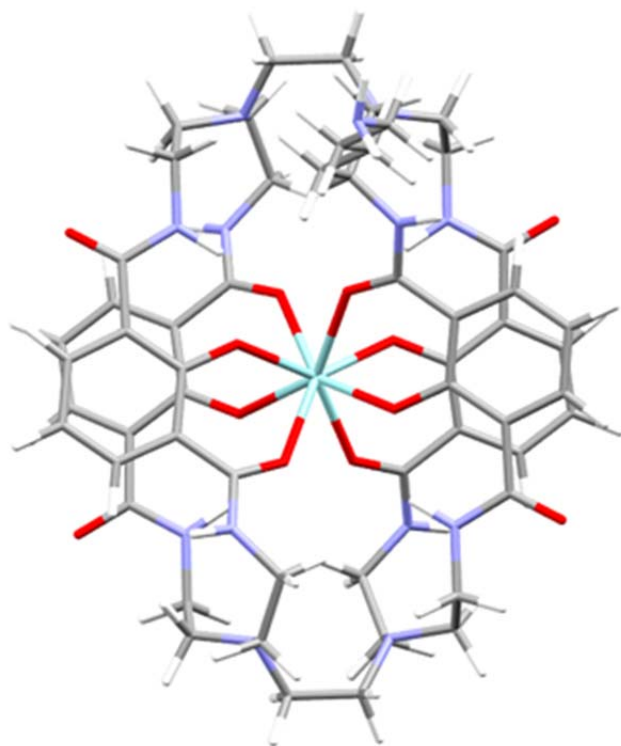

**Zr-1**

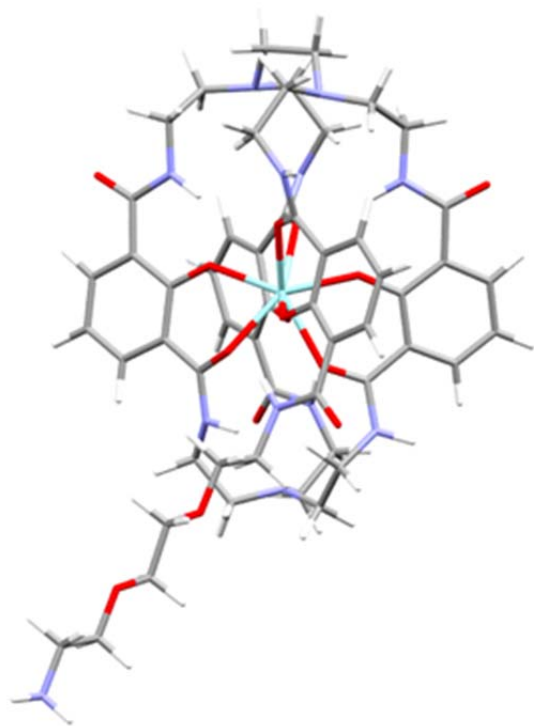

**Zr-2 (Structure A)**

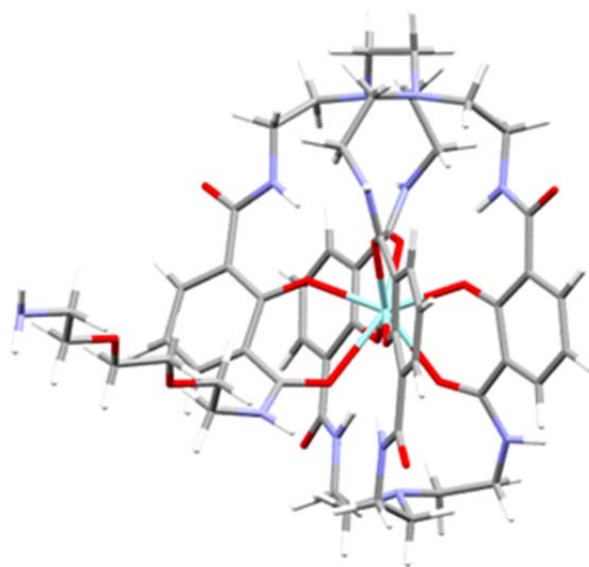

**Zr-2 (Structure B)**

Supplement: S3 Fig — (PDF) [file pone.0178767.s005.pdf]

(A)

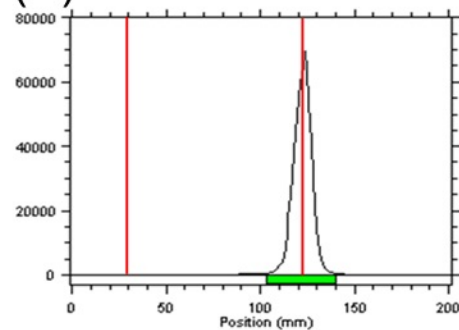

(B)

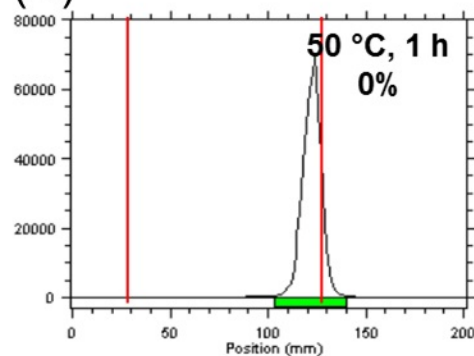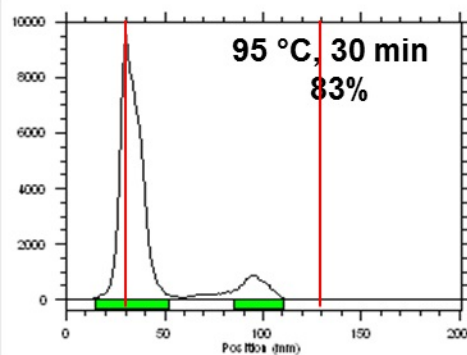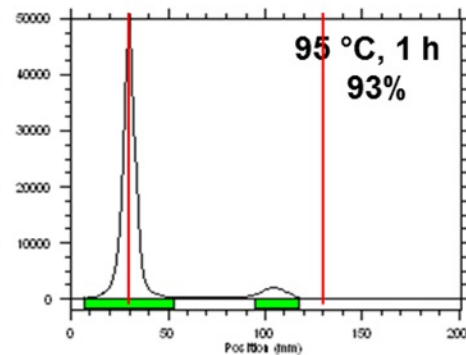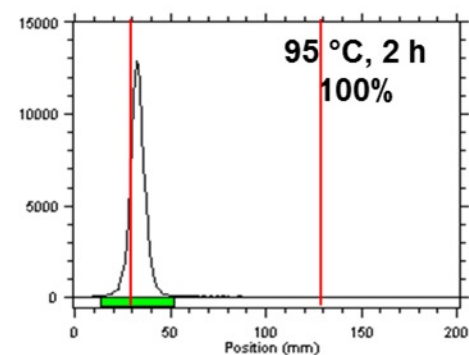

(C)

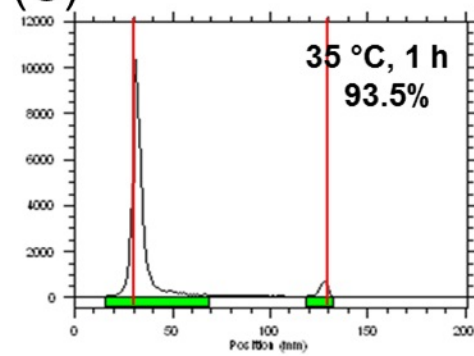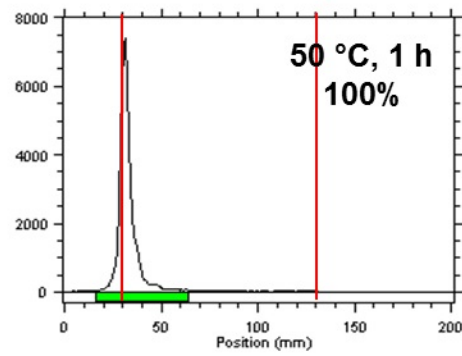

Supplement: S4 Fig — (PDF) [file pone.0178767.s006.pdf]

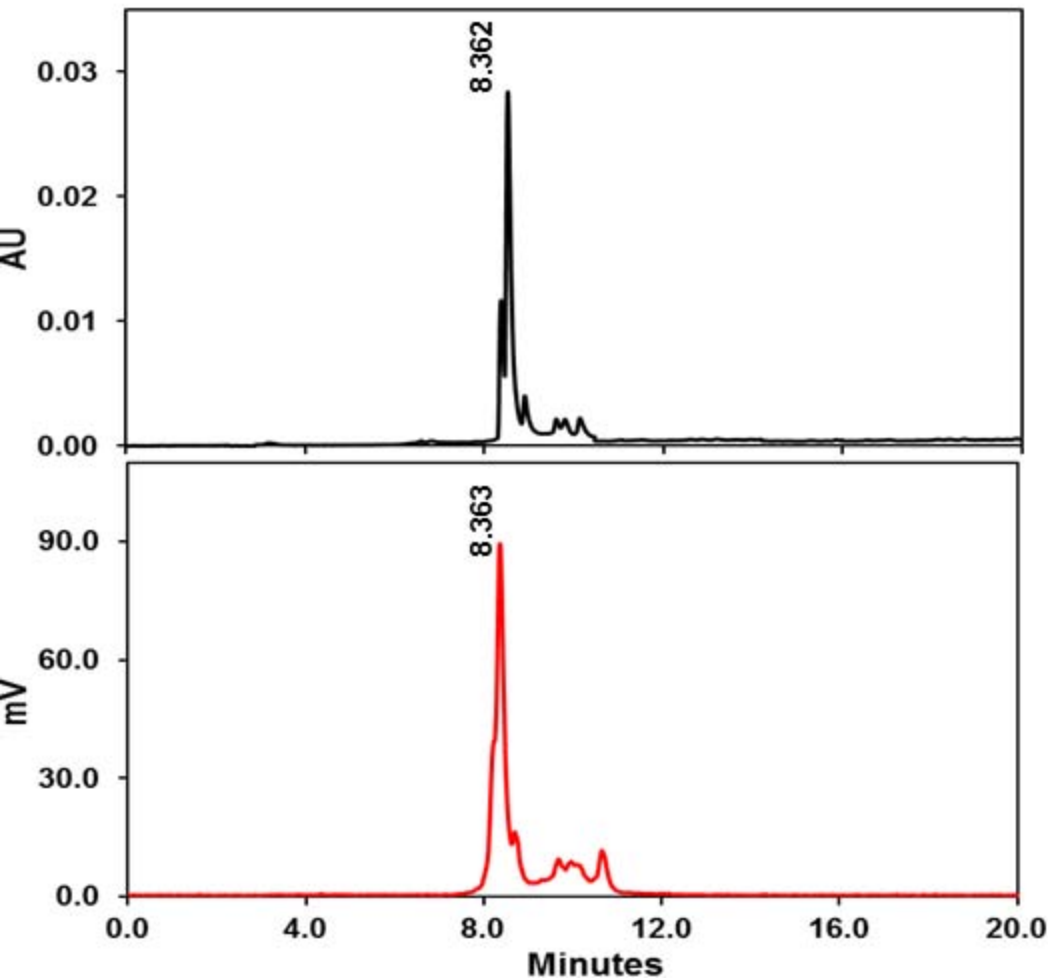

Supplement: S5 Fig — (PDF) [file pone.0178767.s007.pdf]

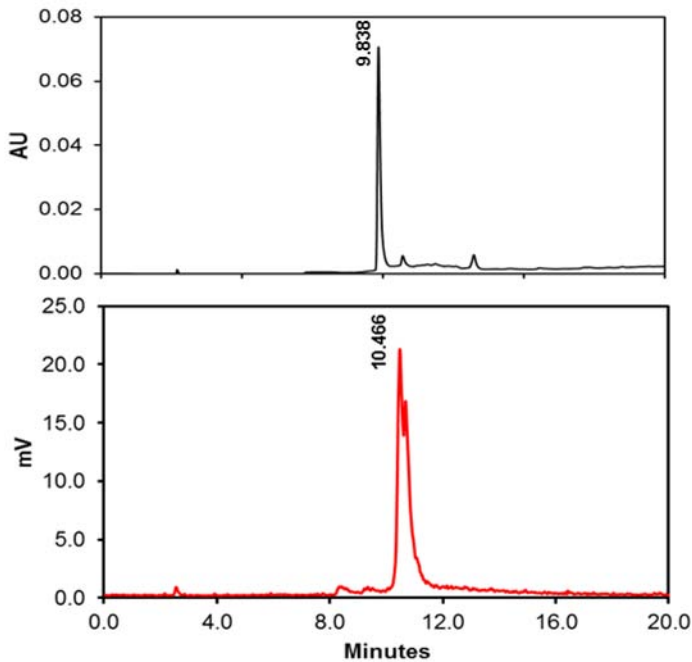

Supplement: S6 Fig — (PDF) [file pone.0178767.s008.pdf]
