# Supplementary material for: Evaluation of macrocyclic hydroxyisophthalamide ligands as chelators for zirconium-89
Source: PLoS One. 2017 Jun 2;12(6):e0178767. doi: 10.1371/journal.pone.0178767 (PMC5456358; doi:10.1371/journal.pone.0178767)
Supplement: S1 Table — (PDF) [file pone.0178767.s009.pdf]

S1 Table. DFT Coordinates for Zr-1 and Zr-2

Zr-1

C	1.268000	2.096000	15.062000
C	-0.968000	1.307000	15.880000
H	-0.606000	0.400000	15.396000
C	-2.252000	1.736000	15.138000
H	-2.529000	2.764000	15.420000
H	-3.067000	1.102000	15.505000
C	-3.262000	0.857000	13.095000
H	-4.241000	1.276000	13.402000
H	-3.219000	0.991000	12.010000
C	-1.904000	2.856000	12.967000
H	-1.306000	3.515000	13.599000
H	-2.856000	3.385000	12.766000
C	-1.152000	2.676000	11.635000
H	-1.491000	1.763000	11.134000
H	-1.393000	3.522000	10.988000
C	1.005000	3.764000	11.384000
C	5.291000	3.835000	11.010000
H	6.372000	3.925000	10.941000
C	5.561000	1.465000	11.740000
N	6.736000	1.400000	11.069000
H	6.886000	2.091000	10.350000
N	0.299000	2.649000	11.734000
H	0.819000	1.812000	11.982000
N	-2.145000	1.596000	13.682000
N	0.091000	2.298000	15.695000
H	-0.035000	3.196000	16.136000
N	6.572000	3.014000	14.735000
H	6.016000	2.164000	14.767000
O	1.550000	0.944000	14.637000
O	6.527000	5.280000	14.643000
O	0.435000	4.821000	11.094000
C	2.159000	3.260000	14.935000
C	1.634000	4.564000	14.976000
H	0.558000	4.713000	14.996000
C	3.837000	5.478000	14.844000
H	4.523000	6.317000	14.808000
C	4.408000	4.206000	14.784000
C	3.566000	3.049000	14.791000
C	5.913000	4.210000	14.703000
C	2.508000	3.673000	11.313000
C	3.126000	4.831000	10.840000
H	2.474000	5.663000	10.600000
C	4.717000	2.639000	11.477000

C	3.306000	2.543000	11.685000
O	4.065000	1.836000	14.681000
O	2.764000	1.455000	12.186000
O	5.248000	0.530000	12.523000
C	1.451000	-1.999000	12.041000
C	-0.641000	-1.176000	10.949000
H	-0.712000	-1.061000	9.859000
H	-0.297000	-0.236000	11.376000
C	-2.027000	-1.524000	11.515000
H	-2.325000	-2.533000	11.180000
H	-2.739000	-0.838000	11.043000
C	-3.288000	-0.644000	13.411000
H	-4.224000	-1.057000	12.982000
H	-3.383000	-0.777000	14.493000
C	-1.988000	-2.666000	13.694000
H	-1.317000	-3.325000	13.140000
H	-2.966000	-3.182000	13.751000
C	-1.425000	-2.517000	15.120000
H	-1.812000	-1.603000	15.581000
H	-1.770000	-3.365000	15.715000
C	0.657000	-3.653000	15.643000
C	4.849000	-3.804000	16.603000
H	5.908000	-3.913000	16.820000
C	5.256000	-1.435000	15.929000
N	6.328000	-1.391000	16.755000
H	6.367000	-2.087000	17.484000
N	0.025000	-2.518000	15.221000
H	0.589000	-1.688000	15.064000
N	-2.111000	-1.393000	12.971000
N	0.371000	-2.179000	11.243000
H	0.298000	-3.068000	10.773000
N	6.642000	-2.999000	13.101000
H	6.109000	-2.141000	12.994000
O	1.681000	-0.855000	12.513000
O	6.551000	-5.272000	13.185000
O	0.035000	-4.704000	15.831000
C	2.298000	-3.177000	12.280000
C	1.765000	-4.473000	12.151000
H	0.701000	-4.605000	11.974000
C	3.913000	-5.420000	12.595000
H	4.573000	-6.271000	12.723000
C	4.487000	-4.158000	12.747000
C	3.671000	-2.988000	12.628000
C	5.966000	-4.186000	13.040000
C	2.137000	-3.591000	15.923000
C	2.665000	-4.763000	16.467000

H	1.972000	-5.585000	16.608000
C	4.364000	-2.595000	16.070000
C	2.997000	-2.475000	15.673000
O	4.166000	-1.784000	12.817000
O	2.546000	-1.374000	15.113000
O	5.068000	-0.494000	15.115000
C	2.460000	5.674000	14.924000
H	2.042000	6.675000	14.929000
C	4.509000	4.934000	10.698000
H	4.965000	5.861000	10.365000
C	4.013000	-4.890000	16.797000
H	4.403000	-5.826000	17.183000
C	2.558000	-5.595000	12.314000
H	2.131000	-6.590000	12.240000
Zr	3.385000	0.033000	13.699000
C	7.759000	0.382000	11.246000
H	7.966000	-0.080000	10.272000
H	7.349000	-0.376000	11.910000
C	9.067000	0.932000	11.838000
H	9.420000	1.787000	11.234000
H	9.824000	0.151000	11.707000
C	10.062000	0.741000	14.059000
H	11.051000	1.009000	13.637000
H	10.025000	1.223000	15.040000
C	8.769000	2.714000	13.511000
H	8.186000	3.144000	12.695000
H	9.739000	3.247000	13.525000
C	8.023000	3.012000	14.827000
H	8.330000	2.301000	15.600000
H	8.304000	4.013000	15.161000
N	8.964000	1.280000	13.255000
C	7.383000	-0.391000	16.724000
H	7.462000	0.066000	17.720000
H	7.080000	0.377000	16.014000
C	8.751000	-0.961000	16.316000
H	9.003000	-1.822000	16.959000
H	9.495000	-0.193000	16.552000
C	10.040000	-0.780000	14.251000
H	10.960000	-1.058000	14.804000
H	10.134000	-1.263000	13.274000
C	8.658000	-2.736000	14.611000
H	7.965000	-3.160000	15.340000
H	9.615000	-3.283000	14.725000
C	8.092000	-3.021000	13.206000
H	8.511000	-2.312000	12.484000
H	8.401000	-4.025000	12.909000

N	8.837000	-1.306000	14.896000
C	-1.192000	1.016000	17.379000
H	-1.922000	0.199000	17.458000
H	-0.253000	0.629000	17.792000
C	-1.674000	2.196000	18.238000
H	-0.956000	3.028000	18.172000
H	-2.627000	2.582000	17.850000
C	-1.849000	1.817000	19.715000
H	-0.904000	1.426000	20.114000
H	-2.578000	1.004000	19.811000
C	-2.306000	2.989000	20.593000
H	-1.582000	3.812000	20.496000
H	-3.256000	3.380000	20.203000
N	-2.466000	2.576000	21.997000
H	-1.788000	3.038000	22.593000
H	-3.382000	2.832000	22.349000

Zr-2 Structure A

C	1.101000	2.455000	15.302000
C	-1.187000	1.868000	16.098000
H	-1.515000	1.999000	17.137000
H	-0.802000	0.856000	15.977000
C	-2.369000	2.091000	15.151000
H	-2.675000	3.151000	15.180000
H	-3.218000	1.533000	15.560000
C	-3.181000	0.802000	13.273000
H	-4.167000	1.296000	13.392000
H	-3.048000	0.673000	12.196000
C	-1.810000	2.790000	12.882000
H	-1.186000	3.500000	13.431000
H	-2.748000	3.323000	12.628000
C	-1.099000	2.463000	11.562000
H	-1.553000	1.579000	11.093000
H	-1.277000	3.286000	10.867000
C	1.094000	2.798000	10.575000
C	5.354000	2.270000	10.094000
H	6.414000	2.131000	9.897000
C	5.695000	1.477000	12.446000
N	7.045000	1.676000	12.348000
H	7.354000	2.285000	11.606000
N	0.353000	2.321000	11.626000
H	0.804000	1.716000	12.303000
N	-2.098000	1.652000	13.776000
N	-0.087000	2.786000	15.850000
H	-0.180000	3.736000	16.178000
O	1.342000	1.249000	15.014000
O	6.586000	5.062000	15.996000
O	0.561000	3.420000	9.651000
C	2.069000	3.552000	15.156000
C	1.681000	4.888000	14.960000
H	0.643000	5.122000	14.734000
C	3.949000	5.600000	15.313000
H	4.699000	6.378000	15.408000
C	4.373000	4.288000	15.524000
C	3.435000	3.222000	15.372000
C	5.802000	4.109000	15.968000
C	2.590000	2.553000	10.523000
C	3.186000	2.924000	9.317000
H	2.531000	3.337000	8.557000

C	4.818000	1.905000	11.342000
C	3.399000	1.996000	11.562000
O	3.798000	1.968000	15.476000
O	2.841000	1.605000	12.682000
O	5.263000	0.941000	13.491000
C	1.314000	-1.809000	12.583000
C	-0.904000	-1.316000	11.513000
H	-1.046000	-1.287000	10.425000
H	-0.689000	-0.309000	11.865000
C	-2.166000	-1.858000	12.203000
H	-2.211000	-2.946000	12.062000
H	-3.047000	-1.459000	11.680000
C	-3.286000	-0.583000	13.937000
H	-4.271000	-1.002000	13.648000
H	-3.325000	-0.462000	15.024000
C	-2.171000	-2.658000	14.552000
H	-1.658000	-3.492000	14.066000
H	-3.194000	-3.009000	14.789000
C	-1.440000	-2.378000	15.880000
H	-1.728000	-1.392000	16.261000
H	-1.761000	-3.127000	16.608000
C	0.661000	-3.542000	16.346000
C	4.968000	-3.774000	16.695000
H	6.040000	-3.866000	16.830000
C	5.280000	-1.443000	15.841000
N	0.012000	-2.457000	15.825000
H	0.579000	-1.675000	15.515000
N	-2.206000	-1.518000	13.626000
N	0.257000	-2.138000	11.804000
H	0.300000	-3.044000	11.363000
N	6.406000	-2.025000	11.828000
H	5.747000	-1.305000	12.103000
O	1.441000	-0.650000	13.047000
O	6.891000	-4.234000	12.061000
O	0.044000	-4.528000	16.761000
C	2.310000	-2.889000	12.730000
C	1.933000	-4.242000	12.681000
H	0.881000	-4.505000	12.757000
C	4.225000	-4.886000	12.444000
H	4.997000	-5.631000	12.280000
C	4.637000	-3.553000	12.490000
C	3.682000	-2.527000	12.762000
C	6.077000	-3.305000	12.144000
C	2.173000	-3.514000	16.420000
C	2.756000	-4.662000	16.960000
H	2.077000	-5.450000	17.268000

C	4.438000	-2.593000	16.147000
C	3.010000	-2.441000	15.985000
O	4.056000	-1.283000	13.005000
O	2.474000	-1.380000	15.423000
O	4.750000	-0.292000	15.724000
C	2.621000	5.910000	15.003000
H	2.325000	6.937000	14.816000
C	4.553000	2.769000	9.081000
H	4.980000	3.035000	8.120000
C	4.138000	-4.807000	17.100000
H	4.555000	-5.713000	17.528000
C	2.885000	-5.247000	12.588000
H	2.589000	-6.291000	12.591000
Zr	3.231000	0.271000	14.270000
C	8.875000	-1.590000	14.870000
H	9.737000	-2.272000	14.798000
H	9.144000	-0.825000	15.609000
C	9.031000	0.442000	13.414000
H	9.546000	0.528000	12.447000
H	9.783000	0.699000	14.176000
C	8.806000	-1.814000	12.441000
H	8.790000	-2.853000	12.775000
H	9.799000	-1.632000	11.990000
N	8.576000	-0.940000	13.593000
C	7.677000	-2.385000	15.437000
H	8.000000	-2.899000	16.351000
H	7.335000	-3.141000	14.721000
C	7.734000	-1.687000	11.355000
H	7.696000	-0.668000	10.961000
H	8.002000	-2.360000	10.532000
H	6.871000	-0.478000	15.417000
N	6.625000	-1.413000	15.723000
C	7.954000	1.550000	13.495000
H	8.478000	2.508000	13.603000
H	7.333000	1.427000	14.382000
H	5.475000	2.116000	16.286000
N	6.158000	2.863000	16.396000
C	7.461000	2.618000	16.982000
H	8.220000	3.186000	16.435000
H	7.692000	1.551000	16.896000
C	7.527000	3.016000	18.458000
H	7.337000	4.095000	18.552000
H	6.751000	2.480000	19.029000
C	9.033000	3.044000	20.280000
H	8.899000	4.128000	20.428000
H	8.325000	2.529000	20.949000

C	10.457000	2.650000	20.644000
H	11.166000	3.173000	19.981000
H	10.594000	1.568000	20.485000
O	8.820000	2.682000	18.930000
C	11.967000	2.690000	22.461000
H	12.727000	3.231000	21.868000
H	12.182000	1.615000	22.360000
C	12.060000	3.096000	23.928000
H	11.742000	4.148000	24.019000
H	11.342000	2.497000	24.498000
O	10.666000	2.999000	21.997000
N	13.407000	2.812000	24.432000
H	14.067000	3.491000	24.060000
H	13.429000	2.919000	25.442000

Zr-2 Structure B

C	2.731000	1.485000	15.740000
C	0.716000	0.687000	16.972000
H	0.851000	0.314000	17.997000
H	0.844000	-0.143000	16.279000
C	-0.708000	1.247000	16.827000
H	-0.784000	2.215000	17.351000
H	-1.367000	0.565000	17.378000
C	-2.443000	0.691000	15.194000
H	-3.226000	1.041000	15.896000
H	-2.788000	0.999000	14.202000
C	-1.153000	2.716000	14.899000
H	-0.372000	3.296000	15.394000
H	-2.111000	3.232000	15.105000
C	-0.876000	2.768000	13.386000
H	-1.391000	1.950000	12.874000
H	-1.271000	3.711000	12.998000
C	1.248000	3.871000	13.041000
C	5.300000	4.096000	11.619000
H	6.334000	4.198000	11.302000
C	5.414000	1.639000	11.132000
N	6.298000	1.912000	10.141000
H	6.248000	2.861000	9.802000
N	0.532000	2.715000	13.034000
H	1.020000	1.848000	12.832000
N	-1.162000	1.352000	15.441000
N	1.783000	1.646000	16.700000
H	1.980000	2.321000	17.423000
N	7.742000	1.359000	13.874000
H	6.908000	0.822000	13.653000
O	2.607000	0.534000	14.929000
O	8.557000	3.271000	14.806000
O	0.737000	4.941000	13.393000
C	3.882000	2.417000	15.757000
C	3.881000	3.598000	16.520000
H	2.968000	3.938000	17.003000
C	6.196000	3.985000	15.994000
H	7.109000	4.564000	16.087000
C	6.250000	2.838000	15.205000
C	5.062000	2.062000	15.033000
C	7.604000	2.517000	14.600000
C	2.673000	3.829000	12.557000

C	3.290000	5.077000	12.462000
H	2.698000	5.934000	12.768000
C	4.713000	2.820000	11.675000
C	3.400000	2.648000	12.211000
O	5.079000	1.033000	14.230000
O	2.886000	1.443000	12.372000
O	5.202000	0.466000	11.521000
C	1.346000	-2.018000	12.035000
C	-0.935000	-1.022000	11.924000
H	-1.390000	-0.754000	10.961000
H	-0.387000	-0.161000	12.302000
C	-2.043000	-1.400000	12.919000
H	-2.555000	-2.318000	12.581000
H	-2.796000	-0.607000	12.857000
C	-2.433000	-0.842000	15.266000
H	-3.486000	-1.179000	15.179000
H	-2.115000	-1.146000	16.267000
C	-1.328000	-2.918000	14.700000
H	-0.973000	-3.478000	13.833000
H	-2.271000	-3.399000	15.027000
C	-0.282000	-3.080000	15.815000
H	-0.409000	-2.295000	16.569000
H	-0.453000	-4.042000	16.303000
C	1.785000	-4.276000	15.336000
C	6.090000	-4.397000	15.251000
H	7.175000	-4.456000	15.251000
C	6.251000	-1.958000	14.687000
N	1.104000	-3.090000	15.378000
H	1.626000	-2.237000	15.208000
N	-1.569000	-1.524000	14.299000
N	0.039000	-2.080000	11.692000
H	-0.250000	-2.872000	11.139000
N	6.564000	-2.958000	11.307000
H	6.051000	-2.177000	11.701000
O	1.783000	-0.963000	12.576000
O	6.454000	-5.012000	10.361000
O	1.209000	-5.357000	15.486000
C	2.182000	-3.188000	11.712000
C	1.622000	-4.464000	11.514000
H	0.567000	-4.633000	11.714000
C	3.779000	-5.340000	10.925000
H	4.423000	-6.154000	10.607000
C	4.387000	-4.104000	11.136000
C	3.587000	-3.000000	11.557000
C	5.879000	-4.060000	10.896000
C	3.283000	-4.221000	15.198000

C	3.939000	-5.431000	15.450000
H	3.308000	-6.291000	15.644000
C	5.469000	-3.168000	14.986000
C	4.050000	-3.058000	14.898000
O	4.131000	-1.823000	11.764000
O	3.485000	-1.902000	14.593000
O	5.952000	-1.225000	13.712000
C	5.018000	4.387000	16.630000
H	4.992000	5.304000	17.209000
C	4.603000	5.226000	12.021000
H	5.074000	6.203000	11.997000
C	5.328000	-5.539000	15.461000
H	5.806000	-6.497000	15.639000
C	2.410000	-5.537000	11.127000
H	1.971000	-6.520000	10.998000
Zr	3.836000	-0.311000	13.191000
C	8.966000	0.778000	11.750000
H	10.011000	0.639000	11.400000
H	8.599000	1.707000	11.310000
C	7.656000	-0.280000	9.932000
H	6.827000	-0.982000	9.832000
H	8.445000	-0.612000	9.226000
C	8.659000	-1.633000	11.683000
H	8.625000	-1.733000	12.772000
H	9.727000	-1.692000	11.392000
N	8.098000	-0.320000	11.323000
C	7.211000	1.069000	9.366000
H	6.758000	0.851000	8.389000
H	8.087000	1.695000	9.165000
C	9.020000	0.993000	13.272000
H	9.713000	1.809000	13.487000
H	9.426000	0.097000	13.760000
C	7.991000	-2.863000	11.055000
H	8.181000	-2.897000	9.975000
H	8.490000	-3.751000	11.461000
H	7.723000	-0.721000	15.130000
N	7.331000	-1.610000	15.430000
C	7.678000	-2.038000	16.782000
H	8.756000	-2.225000	16.842000
H	7.164000	-2.968000	17.016000
C	7.295000	-0.974000	17.814000
H	7.748000	-0.002000	17.552000
H	6.201000	-0.836000	17.817000
C	7.450000	-0.542000	20.133000
H	6.362000	-0.404000	20.229000
H	7.899000	0.449000	19.968000

C	8.005000	-1.153000	21.411000
H	9.094000	-1.290000	21.314000
H	7.560000	-2.149000	21.570000
O	7.758000	-1.425000	19.068000
C	8.155000	-0.724000	23.730000
H	9.254000	-0.836000	23.717000
H	7.730000	-1.707000	23.984000
C	7.745000	0.300000	24.784000
H	8.095000	1.294000	24.458000
H	6.651000	0.344000	24.816000
O	7.690000	-0.274000	22.470000
N	8.233000	-0.127000	26.098000
H	9.239000	0.012000	26.159000
H	7.822000	0.450000	26.826000
