# Supplementary material for: Evaluation of macrocyclic hydroxyisophthalamide ligands as chelators for zirconium-89
Source: PLoS One. 2017 Jun 2;12(6):e0178767. doi: 10.1371/journal.pone.0178767 (PMC5456358; doi:10.1371/journal.pone.0178767)
Supplement: S2 Table — (PDF) [file pone.0178767.s010.pdf]

**Complex**	**Log P (n=8)**
^{89}Zr-1	-2.97 ± 0.02
^{89}Zr-2	-1.45 ± 0.06
^{89}Zr-DFO	-2.83 ± 0.04
