# Supplementary material for: Evaluation of macrocyclic hydroxyisophthalamide ligands as chelators for zirconium-89
Source: PLoS One. 2017 Jun 2;12(6):e0178767. doi: 10.1371/journal.pone.0178767 (PMC5456358; doi:10.1371/journal.pone.0178767)
Supplement: S3 Table — (PDF) [file pone.0178767.s011.pdf]

Day	% intact complex		
	^{89}Zr-1 (n=3)	^{89}Zr-2 (n=3)	^{89}Zr-DFO (n=3)
1	84.7 ± 0.7	64.0 ± 1.2	55.3 ± 0.4
2	83.2 ± 0.5	50.6 ± 1.8	54.2 ± 0.1
3	82.0 ± 0.1	37.9 ± 2.1	53.1 ± 0.3
4	81.3 ± 0.4	36.2 ± 1.4	47.4 ± 0.6
5	75.4 ± 0.6	30.5 ± 1.9	43.7 ± 0.3
6	73.6 ± 0.3	27.4 ± 2.0	42.8 ± 0.4
7	72.2 ± 0.2	27.5 ± 1.6	40.6 ± 0.5
