# Supplementary material for: Evaluation of macrocyclic hydroxyisophthalamide ligands as chelators for zirconium-89
Source: PLoS One. 2017 Jun 2;12(6):e0178767. doi: 10.1371/journal.pone.0178767 (PMC5456358; doi:10.1371/journal.pone.0178767)
Supplement: S4 Table — (PDF) [file pone.0178767.s012.pdf]

Day	^{89}Zr-DFO (n = 3)	^{89}Zr-1 (n = 3)	^{89}Zr-2 (n = 3)
1	100	84.2 ± 0.6	37.0 ± 3.3
2	100	80.5 ± 0.8	20.4 ± 0.8
3	100	80.2 ± 0.9	18.0 ± 1.9
4	100	78.6 ± 0.7	18.9 ± 0.5
5	100	76.7 ± 1.1	17.9 ± 1.0
6	100	75.8 ± 1.2	16.3 ± 0.9
7	100	75.1 ± 0.4	17.0 ± 0.6
