# Supplementary material for: Evaluation of macrocyclic hydroxyisophthalamide ligands as chelators for zirconium-89
Source: PLoS One. 2017 Jun 2;12(6):e0178767. doi: 10.1371/journal.pone.0178767 (PMC5456358; doi:10.1371/journal.pone.0178767)
Supplement: S5 Table — (PDF) [file pone.0178767.s013.pdf]

Tissue/Organ	2 h	4 h	24 h	48 h	72 h
Blood	0.092 ± 0.028	0.024 ± 0.009	0.003 ± 0.000	0.001 ± 0.001	0.001 ± 0.001
Heart	0.101 ± 0.030	0.056 ± 0.013	0.025 ± 0.004	0.023 ± 0.004	0.023 ± 0.005
Lung	0.332 ± 0.105	0.233 ± 0.041	0.074 ± 0.010	0.053 ± 0.008	0.045 ± 0.004
Liver	0.509 ± 0.088	0.523 ± 0.044	0.377 ± 0.036	0.324 ± 0.022	0.261 ± 0.019
Small intestine	0.299 ± 0.066	0.136 ± 0.031	0.041 ± 0.011	0.029 ± 0.003	0.019 ± 0.001
Large intestine	0.527 ± 0.075	0.598 ± 0.065	0.072 ± 0.016	0.039 ± 0.003	0.022 ± 0.003
Kidney	16.128 ± 2.964	16.826 ± 2.123	10.658 ± 1.820	6.735 ± 1.232	4.709 ± 0.837
Spleen	0.172 ± 0.033	0.169 ± 0.037	0.120 ± 0.021	0.110 ± 0.024	0.091 ± 0.015
Pancreas	0.047 ± 0.012	0.034 ± 0.008	0.021 ± 0.003	0.019 ± 0.003	0.016 ± 0.002
Stomach	0.152 ± 0.034	0.049 ± 0.012	0.032 ± 0.012	0.018 ± 0.005	0.011 ± 0.001
Muscle	0.053 ± 0.022	0.030 ± 0.011	0.008 ± 0.004	0.016 ± 0.005	0.012 ± 0.004
Fat	0.025 ± 0.012	0.016 ± 0.012	0.007 ± 0.003	0.009 ± 0.006	0.009 ± 0.002
Bone	0.205 ± 0.089	0.136 ± 0.039	0.107 ± 0.019	0.117 ± 0.012	0.107 ± 0.006
