# Supplementary material for: Evaluation of macrocyclic hydroxyisophthalamide ligands as chelators for zirconium-89
Source: PLoS One. 2017 Jun 2;12(6):e0178767. doi: 10.1371/journal.pone.0178767 (PMC5456358; doi:10.1371/journal.pone.0178767)
Supplement: S6 Table — (PDF) [file pone.0178767.s014.pdf]

Tissue/Organ	2 h	4 h	24 h	48 h	72 h
Blood	0.279 ± 0.066	0.144 ± 0.041	0.011 ± 0.006	0.003 ± 0.002	0.002 ± 0.001
heart	0.149 ± 0.018	0.081 ± 0.009	0.033 ± 0.005	0.027 ± 0.006	0.030 ± 0.003
lung	0.500 ± 0.106	0.281 ± 0.084	0.104 ± 0.010	0.081 ± 0.009	0.073 ± 0.007
liver	2.035 ± 0.549	1.612 ± 0.112	1.521 ± 0.152	1.434 ± 0.105	1.437 ± 0.148
Small intestine	1.797 ± 0.618	0.421 ± 0.154	0.039 ± 0.007	0.028 ± 0.004	0.023 ± 0.004
Large intestine	5.120 ± 2.489	5.731 ± 2.734	0.153 ± 0.058	0.033 ± 0.004	0.031 ± 0.006
kidney	7.964 ± 1.030	6.893 ± 0.782	4.950 ± 0.425	4.025 ± 0.995	3.598 ± 0.789
spleen	0.325 ± 0.059	0.247 ± 0.029	0.226 ± 0.032	0.230 ± 0.026	0.227 ± 0.031
pancreas	0.087 ± 0.035	0.061 ± 0.010	0.031 ± 0.004	0.030 ± 0.006	0.028 ± 0.011
stomach	0.168 ± 0.088	0.131 ± 0.052	0.037 ± 0.019	0.020 ± 0.006	0.016 ± 0.004
muscle	0.086 ± 0.022	0.040 ± 0.003	0.019 ± 0.003	0.012 ± 0.002	0.014 ± 0.004
fat	0.121 ± 0.087	0.042 ± 0.014	0.032 ± 0.018	0.026 ± 0.009	0.028 ± 0.013
bone	0.427 ± 0.161	0.503 ± 0.083	0.954 ± 0.167	0.975 ± 0.103	0.679 ± 0.333
