# Supplementary material for: Evaluation of macrocyclic hydroxyisophthalamide ligands as chelators for zirconium-89
Source: PLoS One. 2017 Jun 2;12(6):e0178767. doi: 10.1371/journal.pone.0178767 (PMC5456358; doi:10.1371/journal.pone.0178767)
Supplement: S7 Table — (PDF) [file pone.0178767.s015.pdf]

Tissue/Organ	2 h	4 h	24 h	48 h	72 h
Blood	0.009 ± 0.003	0.005 ± 0.001	0.001 ± 0.001	0.001 ± 0.001	0.000 ± 0.001
Heart	0.020 ± 0.003	0.019 ± 0.003	0.014 ± 0.002	0.010 ± 0.002	0.009 ± 0.004
Lung	0.060 ± 0.009	0.038 ± 0.006	0.024 ± 0.006	0.019 ± 0.005	0.017 ± 0.004
Liver	0.234 ± 0.023	0.163 ± 0.051	0.081 ± 0.012	0.070 ± 0.007	0.066 ± 0.009
Small intestine	0.357 ± 0.175	0.130 ± 0.080	0.013 ± 0.002	0.008 ± 0.001	0.006 ± 0.001
Large intestine	0.877 ± 0.435	1.020 ± 0.207	0.024 ± 0.004	0.009 ± 0.002	0.008 ± 0.001
Kidney	2.051 ± 0.238	1.848 ± 0.382	1.340 ± 0.137	0.957 ± 0.216	0.689 ± 0.098
Spleen	0.037 ± 0.005	0.036 ± 0.004	0.036 ± 0.007	0.030 ± 0.008	0.027 ± 0.007
Pancreas	0.015 ± 0.005	0.013 ± 0.002	0.012 ± 0.002	0.009 ± 0.003	0.007 ± 0.002
Stomach	0.140 ± 0.124	0.055 ± 0.038	0.014 ± 0.005	0.005 ± 0.003	0.005 ± 0.002
Muscle	0.011 ± 0.001	0.008 ± 0.003	0.006 ± 0.002	0.004 ± 0.001	0.004 ± 0.002
Fat	0.013 ± 0.003	0.009 ± 0.002	0.007 ± 0.002	0.005 ± 0.008	0.008 ± 0.004
Bone	0.051 ± 0.017	0.058 ± 0.008	0.082 ± 0.016	0.092 ± 0.011	0.078 ± 0.014
